# Sustaining control of schistosomiasis mansoni in moderate endemicity areas in western Côte d’Ivoire: a SCORE study protocol

**DOI:** 10.1186/1471-2458-14-1290

**Published:** 2014-12-17

**Authors:** Rufin K Assaré, Stefanie Knopp, Nicaise A N’Guessan, Ahoua Yapi, Yves-Nathan T Tian-Bi, Patrick K Yao, Jean T Coulibaly, Mamadou Ouattara, Aboulaye Meïté, Alan Fenwick, Eliézer K N’Goran, Jürg Utzinger

**Affiliations:** Department of Epidemiology and Public Health, Swiss Tropical and Public Health Institute, P.O. Box, Basel, CH–4002 Switzerland; University of Basel, P.O. Box, Basel, CH–4003 Switzerland; Unité de Formation et de Recherche Biosciences, Université Félix Houphouët-Boigny, 22 BP 770, Abidjan, 22 Côte d’Ivoire; Centre Suisse de Recherches Scientifiques en Côte d’Ivoire, 01 BP 1303, Abidjan, 01 Côte d’Ivoire; Wolfson Wellcome Biomedical Laboratories, Department of Life Sciences, Natural History Museum, Cromwell Road, London, SW7 5BD UK; Programme National de Lutte contre la Schistosomiase, les Géohelminthiases et la Filariose Lymphatique, Ministère de la Santé et de l’Hygiène Publique, 06 BP 6394, Abidjan, 06 Côte d’Ivoire; Schistosomiasis Control Initiative, Department of Infectious Disease Epidemiology, Faculty of Medicine, Imperial College London, VB1 Norfolk Place, St. Mary’s Campus, London, W2 1PG UK

**Keywords:** Schistosomiasis, *Schistosoma mansoni*, Control, Morbidity control, Preventive chemotherapy, Praziquantel, Prevalence, Intensity of infection, Côte d’Ivoire, SCORE

## Abstract

**Background:**

Schistosomiasis is a parasitic disease that occurs in the tropics and subtropics. The mainstay of control is preventive chemotherapy with praziquantel. In Africa, an estimated 230 million people require preventive chemotherapy. In western Côte d’Ivoire, infections with *Schistosoma mansoni* are widespread. To provide an evidence-base for programme decisions about preventive chemotherapy to sustain control of schistosomiasis, a 5-year multi-country study with different treatment arms has been designed by the Schistosomiasis Consortium for Operational Research and Evaluation (SCORE) and is currently being implemented in various African settings, including Côte d’Ivoire.

**Methods/Design:**

We report the study protocol, including ethics statement and insight from a large-scale eligibility survey carried out in four provinces in western Côte d’Ivoire. The study protocol has been approved by the ethics committees of Basel and Côte d’Ivoire. A total of 12,110 children, aged 13–14 years, from 264 villages were screened for *S. mansoni* using duplicate Kato-Katz thick smears from single stool samples. Among the schools with a *S. mansoni* prevalence of 10-24%, 75 schools were selected and randomly assigned to one of three treatment arms. In each school, three stool samples are being collected from 100 children aged 9–12 years annually and one stool sample from 100 first-year students at baseline and in the final year and subjected to duplicate Kato-Katz thick smears. Cost and coverage data for the different intervention arms, along with environmental, political and other characteristics that might impact on the infection prevalence and intensity will be recorded in each study year, using a pretested village inventory form.

**Discussion:**

The study will document changes in *S. mansoni* infection prevalence and intensity according to different treatment schemes. Moreover, factors that determine the effectiveness of preventive chemotherapy will be identified. These factors will help to develop reasonable measures of force of transmission that can be used to make decisions about the most cost-effective means of lowering prevalence, intensity and transmission in a given setting. The gathered information and results will inform how to effectively sustain control of schistosomiasis at a low level in different social-ecological contexts.

**Trial registration:**

ISRCTN99401114 (date assigned: 12 November 2014).

## Background

### Burden and transmission of schistosomiasis, with an emphasis on *Schistosoma mansoni*in Africa

Human schistosomiasis, a disease caused by chronic infection with parasitic trematodes of the genus *Schistosoma*, is endemic in 78 tropical and subtropical countries, 42 of which are located in Africa [1]. An estimated 779 million people are at risk of schistosomiasis, more than 230 million are infected, 120 million are symptomatic and 20 million suffer from severe and debilitating forms of schistosomiasis [2–5]. The burden of the disease is essentially concentrated in Africa, where more than 90% of the infections worldwide occur [5–7]. Schistosomiasis is intimately connected with poverty, and hence, the disease delays the social and economic development in endemic countries [5, 8–10].

The life cycle of schistosomiasis involves a phase of sexual reproduction by adult schistosome worms in the definitive human host, and an asexual phase in the intermediate host, a specific freshwater snail. In Côte d’Ivoire, for example, *Biomphalaria pfeifferi* is the only intermediate host snail for *Schistosoma mansoni* [11]. From the snail, cercariae are released into the surrounding water and can invade humans through the skin. Infection with *S. mansoni* causes intestinal schistosomiasis. Typical symptoms include blood in the stool, (bloody) diarrhoea, chronic or intermittent abdominal pain, anaemia, general fatigue, weight loss, hepatomegaly, splenomegaly and marked eosinophilia [8, 12]. Moreover, chronic infection can impair children’s physical and cognitive development and nutritional status [13]. Associations of intestinal schistosomiasis with hepatitis, acquired immunodeficiency syndrome (AIDS) and malaria hypertension have been reported [14–16].

Several factors contribute to the spread of schistosomiasis. Demographic features, including age, gender, ethnicity and socioeconomic status have a strong influence on the spatial distribution of schistosomiasis, particularly *S. mansoni* [17, 18] *.* Tourism, construction and operation of water resource developments (i.e. irrigation schemes and dams) are associated with higher risks of *S. mansoni*, explained by the creation of favourable conditions for intermediate host snails and higher frequencies of human-water contacts [5, 19, 20]. It is estimated that more than 100 million people at risk of schistosomiasis live in irrigation schemes or in close proximity to reservoirs of large dams [5]. Intense rainfall and flooding might be responsible for the reintroduction of intermediate host snails to areas from which schistosomiasis had previously been eliminated [21].

### Schistosomiasis control in Africa

According to the World Health Organization (WHO), comprehensive schistosomiasis control programmes should include treatment of at-risk groups, provision of clean water, adequate sanitation, hygiene education and snail control [1]. The current mainstay of control is preventive chemotherapy – that is the periodic administration of the antischistosomal drug praziquantel to entire at-risk populations without prior diagnosis. The goal is to cover at least 75% of those at risk of schistosomiasis by preventive chemotherapy in 2020 [7]. The frequency of preventive chemotherapy is guided by infection prevalence in specific age groups. In areas where the prevalence of *Schistosoma* infection in school-aged children (5–14 years) is 50% or higher, entire communities should be treated once every year; if the prevalence is between 10% and 50%, treatment is focussed on school-aged children with a frequency once every two years; if the prevalence is below 10%, school-aged children should be treated twice, at school entry and again before they finish schooling [22, 23]. In Africa, approximately 35.5 million people were treated with praziquantel in 2012 [1]. This estimate accounts for a coverage of only 13.6% of school-aged children. Hence, concerted efforts are needed to massively scale-up preventive chemotherapy to reach the 75% coverage goal by the year 2020. In Côte d’Ivoire, where both *S. mansoni* and *S. haematobium* are endemic and many people suffer from intestinal or urogenital schistosomiasis [17, 24–29], no large-scale preventive chemotherapy programme was in place prior to the onset of this study in 2011 [28, 30, 31].

### Operational research for schistosomiasis control

Further up-scaling of schistosomiasis control in the years to come will not only need political commitment, national strategic plans, dedicated development partners, functioning health systems and community volunteers, but will also involve major costs. To assess which strategy of preventive chemotherapy will provide the best balance in terms of reduction in prevalence and intensity of schistosome infection in school-aged children on one hand, and costs on the other hand, the Schistosomiasis Consortium for Operational Research and Evaluation (SCORE; http://score.uga.edu/) designed a series of large-scale, multi-country intervention studies. In response to a request for proposals to gain and sustain control of schistosomiasis in Africa, several investigators from Africa, in partnership with colleagues from Europe and the United States of America, put forward their ideas. The proposals were evaluated by a panel of experts against predefined criteria. Initially, three projects were selected under the heading “Sustaining control of schistosomiasis”, focusing either on *S. mansoni* (two projects; Côte d’Ivoire and Kenya) or *S. haematobium* (one project; Niger). The overarching goal is to evaluate alternative approaches to preventive chemotherapy in areas with moderate prevalence of infection at baseline (10-24% in school-aged children). Of note, the sustaining schistosomiasis haematobia project in Niger has been terminated after two years due to an issue with the randomisation of study villages.

Here, we summarise the relevant part of a harmonised study protocol that is being followed by partners conducting the sustaining control of schistosomiasis mansoni studies in Côte d’Ivoire and Kenya. The field and laboratory procedures for the sustaining *S. mansoni* control project in moderate endemicity areas of Côte d’Ivoire will be presented in greater detail, including results of the initial eligibility survey, which guided the selection of 75 communities or villages for subsequent treatment interventions.

### Goal, aims and objectives

The goal of the SCORE projects aiming at sustaining control of schistosomiasis at a low level is to generate an evidence-base for programme decisions about preventive chemotherapy-based approaches to sustain the control of *S. mansoni* infections. The studies will determine which strategy for preventive chemotherapy provides the best balance in terms of cost and the reduction in prevalence and intensity of infection in school-aged children after four years of intervention. The studies are designed to answer the following question: How can we sustain control of *S. mansoni* in communities/villages with a moderate endemicity level (prevalence of 10-24%, as assessed by a single stool examination with duplicate Kato-Katz thick smears)? Specifically, we are addressing the following research questions:

- ● What combination of annual school-based treatment (SBT) and “drug holidays” yields the best outcomes for the lowest cost?
- ● What are the factors that determine the effectiveness of preventive chemotherapy?
- ● Can reasonable measures of force of transmission be developed that can be utilised to make decisions about the most cost-effective means of lowering prevalence, intensity and transmission in a given setting?

## Methods/Design

### Study design

**Figure 1:**
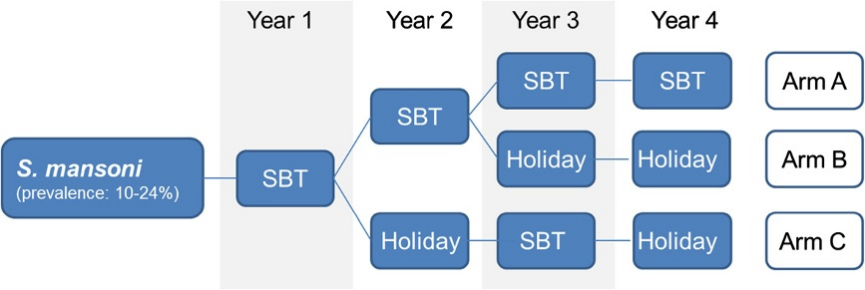
**Study arms for the sustaining control of** ***Schistosoma mansoni*** **studies in moderate endemicity areas (prevalence: 10-24%).** SBT, school-based treatment; Holiday, no drug delivery.

The SCORE sustaining schistosomiasis control studies are designed as randomised intervention trials with three study arms. Each arm comprises 25 communities or villages. Hence, the studies will include 75 communities per country. Communities will be provided with various combinations of SBT and “drug holidays” over a 4-year period, followed by final data collection, analysis and dissemination of results in the fifth year. The intervention arms in the sustaining schistosomiasis control studies are designed as shown in Figure 1. In brief:

- ● schools of arm A will receive annual SBT for four years;
- ● schools of arm B will receive SBT in the first two years, followed by “drug holidays”; and
- ● schools of arm C will receive SBT in years 1 and 3, alternated by “drug holidays” in years 2 and 4.

Preventive chemotherapy with praziquantel is being provided as single oral dose of 40 mg/kg, using a dose pole. Standard praziquantel treatment exclusion criteria apply [22]. No treatment will be provided during “drug holidays” and no parasitological data are collected in those years. During SBT, praziquantel will be administered by trained teachers to all school-going children. Children in all schools in the community will be treated, and hence, children attending schools not otherwise involved in the study will also receive treatment. Any time SBT will take place, additional efforts will be made to enhance treatment coverage, such as community sensitization and mobilization efforts, radio announcements, and other means of information, education and communication (IEC) strategies. Non-school attendees who span the same age range as school-going children will also be invited for treatment. No other major treatment strategies outside the education sector venue will be implemented. The school attendance rates and treatment coverage will be documented throughout the study.

### Justification of the number of intervention arms and participants

The protocol for the sustaining schistosomiasis control studies was developed through a series of expert consultations, facilitated by the SCORE secretariat and its scientific advisory board. The decision to choose three intervention arms for the current studies took into consideration formal sample size calculation and operational feasibility. For sample size calculation, it was assumed that the treatment interventions will reduce *Schistosoma* prevalence in moderate endemicity areas from 25% to 10%. Analyses determined the minimum effect size that may be detected with 90% power for a 2-sided *α* = 0.05 level test as a function of the number of children *m* tested per village, the number *n* of villages sampled per treatment, the overdispersion parameter *φ*, and the correlation *ρ* between observations in year 1 at baseline and in year 4 at the end of the study. The calculations revealed that studying 20 communities or schools per arm and evaluating 100 individuals per school would result in minimum effect sizes of 5-12% with or without overdispersion. This minimum effect size was deemed reasonable. To further increase the chance of detecting differences between the interventions arms, the number of the units of interventions, and hence, the number of schools, was increased to 25 per arm. Taken together, the trial protocol asked to examine 100 children aged 9–12 years every year whenever drug intervention will be implemented as primary outcome. Additionally, 100 first-year students will be examined in years 1 and 5.

### Eligibility of study communities

Sustaining schistosomiasis control studies will include 75 communities with an initial *S. mansoni* prevalence of 10-24%. Selection of these large numbers of communities has been determined through a rapid appraisal eligibility survey. A single stool sample from each of 50 children aged 13–14 years has been subjected to duplicate Kato-Katz thick smears [32, 33]. Additional criteria applied for eligibility determination are as follows. First, a study community must have a primary school, because the arms of the study are school-based and every participating community must be eligible to be randomised to any of the study arms. However, it is permitted that a study community may have more than one school. If two nearby communities have schools with less than 100 children per school but they are similar in terms of ecology and socioeconomic status, they can be combined for purposes of this study and be considered as a single study community. Second, two nearby communities that share water sources and/or whose schools have overlapping catchment areas will not be considered separate villages for the purposes of this study; one of the two schools will be chosen. Third, there is no pre-set population requirement for the size of a community, as long as it includes at least 100 children aged 9–12 years who attend school. Fourth, preference is for settings that have not recently been subjected to preventive chemotherapy targeting schistosomiasis. If communities have been previously treated, historic treatment data should be included where available. Fifth and finally, to the extent possible, study communities should be as similar as possible in characteristics that could affect transmission dynamics, including, for example, a history of past treatment and the availability of water sources.

### Eligibility of study participants

For the eligibility study to rapidly identify the 75 communities with a baseline prevalence of 10-24%, children were eligible to participate if they were 13 or 14 years old and provided an informed consent sheet signed by their parents. In the baseline and the yearly follow-up surveys conducted to assess the change in prevalence and intensity of *Schistosoma* infection in each intervention arm, children aged 9–12 years who provide a written informed consent from their parents will be included. Additionally, in years 1 and 5, first-year students will provide written informed consent from their parents and will participate in the study.

### Details of the sustaining *S. mansoni*control study in western Côte d’Ivoire

#### Study area and population

The sustaining *S. mansoni* control study in Côte d’Ivoire is being conducted by a team of researchers from the Université Félix Houphouët-Boigny, who work in close collaboration with the Programme National de Lutte contre la Schistosomiase, les Géohelminthiases et la Filariose Lymphatique (PNL-SGF) at the Ministry of Health and Public Hygiene, the ‘Centre Suisse de Recherches Scientifiques en Côte d’Ivoire (CSRS), all based in Abidjan, and the Swiss Tropical and Public Health Institute (Swiss TPH) in Basel, Switzerland.

**Figure 2:**
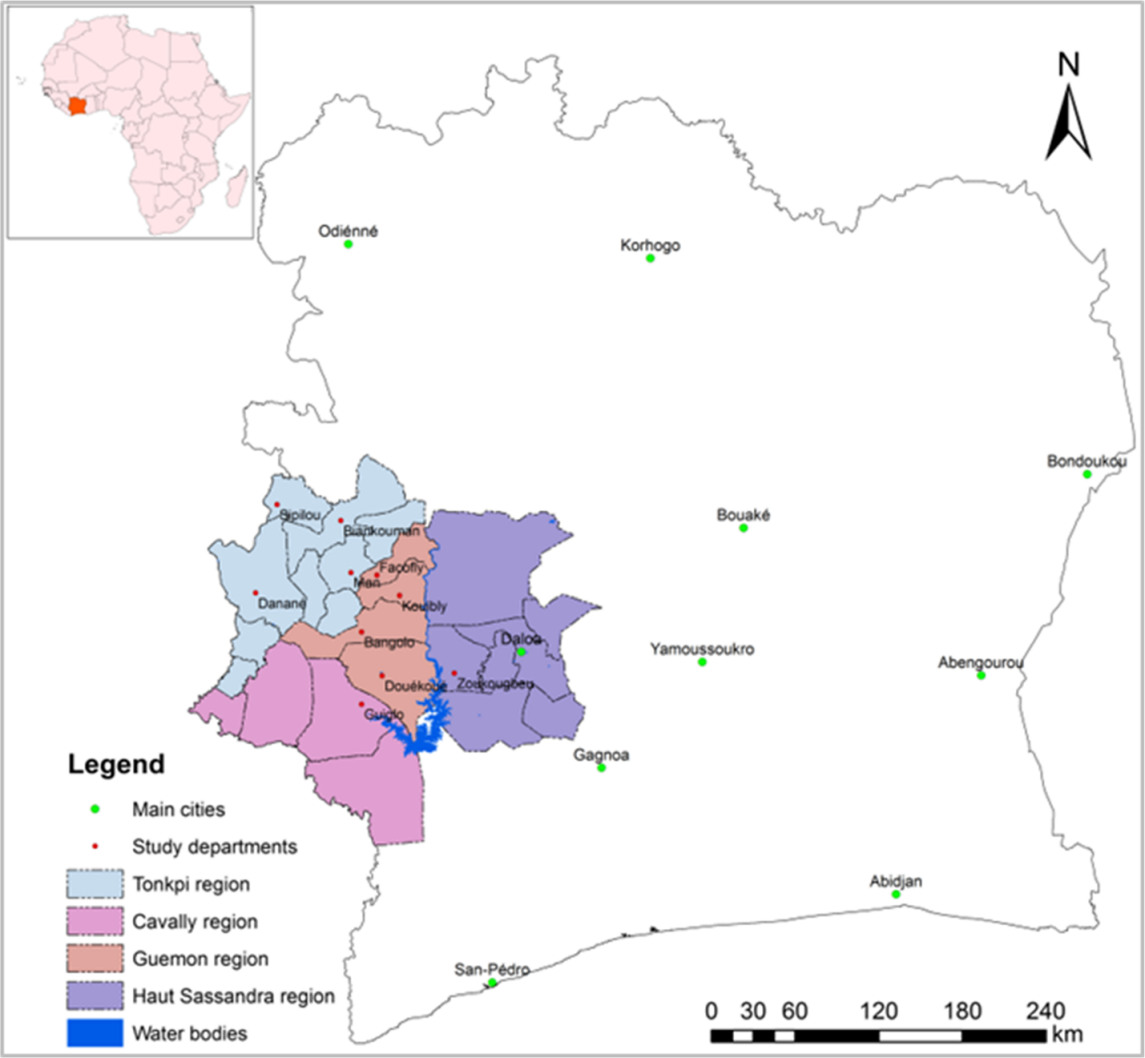
**Map of Côte d’Ivoire with the four study regions in the western part where the SCORE sustaining** ***S. mansoni*** **control project is being implemented.**

The study area is located in western Côte d’Ivoire in the four regions Cavaly, Guemon, Haut-Sassandra and Tonkpi (Figure 2). The area was chosen because it is a well known *S. mansoni* focus [25] and our teams conducted schistosomiasis research there since the mid-1990s, including treatment of individuals found positive upon Kato-Katz examination [17, 24, 28, 29, 34–37]. Cavaly, Guemon and Tonkpi regions are located west of the Sassandra River and belong to the district des Montagnes. Ten departments of this district are included in our study: Bangolo, Biankouma, Danané, Douékoué, Facobly, Guiglo, Kouibly, Man, Sipilou and Zoukougbeu. The district des Montagnes is a mountainous area with an average altitude ranging between 300 m and slightly above 1,000 m. The climate is humid tropical with two seasons. The rainy season usually lasts from March to October. The Haut-Sassandra region is located East of the Sassandra River. Zoukougbeu is the only department of that region which is included in our study. Here, the average altitude ranges between 200 m and 300 m. The climate is sub-equatorial, characterised by two rainy seasons. The long rainy season occurs from March to July and the short rainy season from September to October.

According to the national population census carried out in 1998 (the most recent census at the time of writing the current piece), the total population in the study area is 1.5 million people (unpublished data; Institut National de la Statistique en Côte d’Ivoire). Most people belong to one among the four ethnic groups: Bété, Guéré, Wobé and Yacouba. People are mainly engaged in subsidence farming (cassava, maize, plantain and rice). There is also production of cash crops (coffee and cocoa) and a small forestry industry in the town of Man [38].

The annual rainfall in the study area varies between 1,100 and 2,000 mm. The vegetation is composed of two types of forests (semi-deciduous and evergreen mountain forest). The average annual temperature is around 26°C.

#### Selection and randomisation of study villages

The 75 villages with a *S. mansoni* prevalence of 10-24% in western Côte d’Ivoire were identified as follows. First, our team organised a series of meetings with health and education authorities in the four study regions. The purpose and procedures of the study were explained and a total of 264 communities fulfilling the following criteria were identified: (i) village has a school attended by at least 100 children aged 9–12 years in grades 2–5 and 50 children aged at least 13 years in grades 4–6; (ii) village and school had no recent history of preventive chemotherapy using praziquantel against schistosomiasis (within the past 12 months); (iii) village is accessible also in the rainy season; and (iv) it is safe for our teams to work in the village. The latter issue was a real concern, as Côte d’Ivoire suffered from a decade-long political unrest that culminated in armed conflict and war in late 2010/early 2011 [39]. Before the onset of the surveys in each school, we conducted a brief interview with the school teachers to assess the selectability of the villages, according to the aforementioned criteria. Then, schoolchildren in grades 1–6 were informed about the mode of transmission of *S. mansoni*, its health impact and the importance of the current project. Children aged 13 or 14 years were randomly selected from grades 4–5 until the number of children reached 50. In settings where less than 50 children aged 13–14 years were present, the sample was completed with 12-year-old children, but these children will not be enrolled in the subsequent randomised controlled trial.

Children were asked to provide a written informed consent from their parents or legal guardians. Children with written informed consent were supplied with plastic containers to collect a small amount of their own early morning stool specimen. Collection containers were labelled with unique identification numbers. The name, sex, age and school grade of each child were recorded.

Stool samples were transferred to the Centre Hospitalier Regional de Man for parasitological examination. From each stool sample, duplicate Kato-Katz thick smears were prepared on microscope slides using 41.7 mg templates [32, 33]. After allowing the slides to clear for at least 60 min, they were examined under a microscope by experienced laboratory technicians for the presence of *S. mansoni* and soil-transmitted helminths (i.e. *Ascaris lumbricoides*, hookworm and *Trichuris trichiura*).

**Figure 3:**
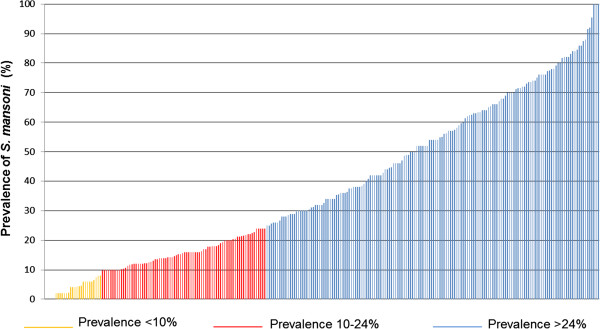
**Range of prevalence of** ***S. mansoni*** **infection in the 264 villages screened in western Côte d’Ivoire to identify moderate** ***S. mansoni*** **endemicity areas (10-24%).** At the unit of the school, we examined 50 children aged 13–14 years with duplicate Kato-Katz thick smears from a single stool sample.

**Figure 4:**
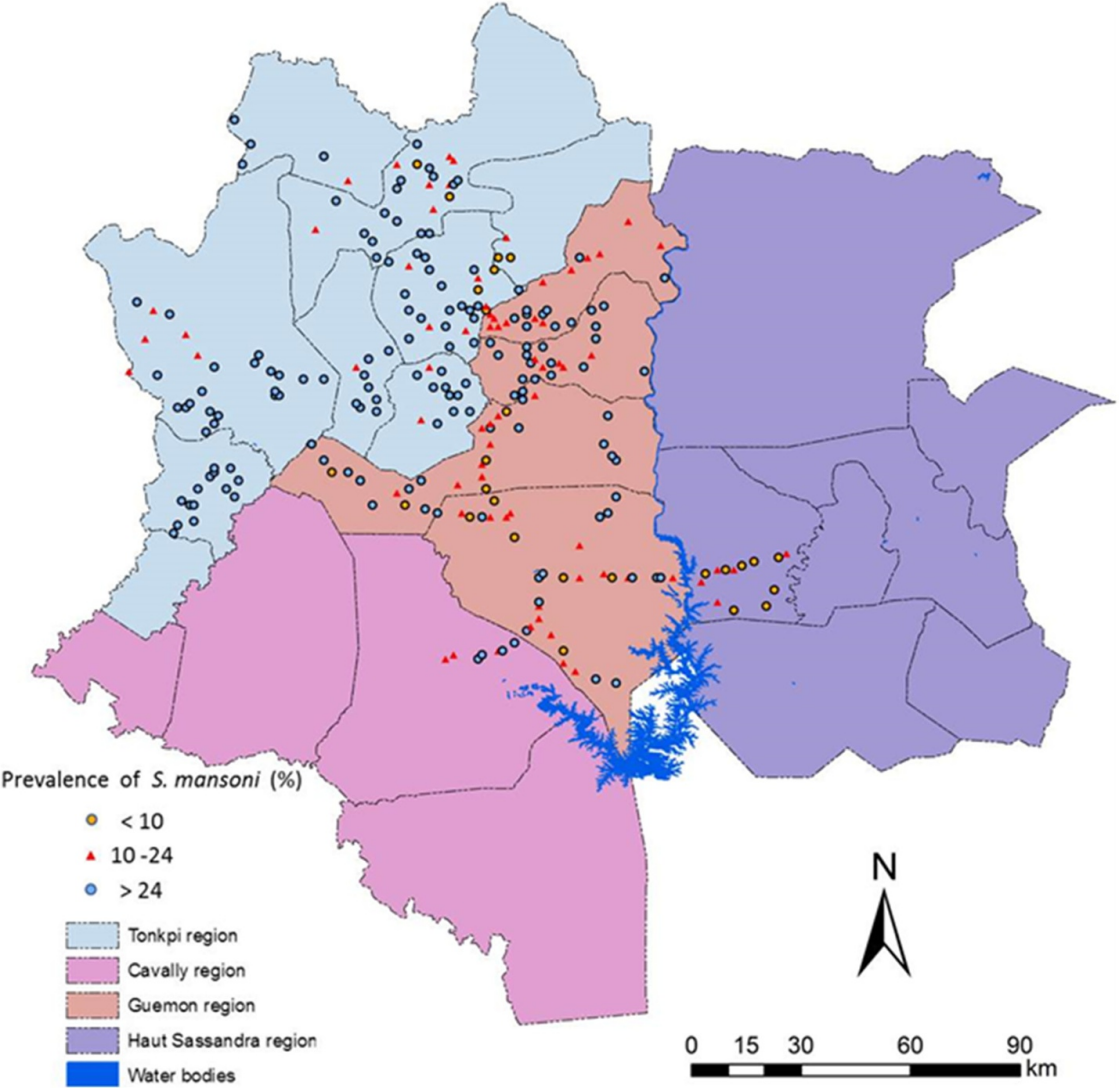
**Map showing point prevalence of** ***S. mansoni*** **in 264 schools of western Côte d’Ivoire, as determined by an eligibility survey in late 2011/early 2012.**

The prevalence of *S. mansoni* was calculated for each school. A total of 12,110 children submitted a stool sample that was subjected to duplicate Kato-Katz thick smears. The prevalence at the unit of the school in the eligibility survey ranged from 0% to 100% (Figure 3). In brief, among the 264 schools, 157 (59.5%) had a *S. mansoni* prevalence above 24%, 78 (29.5%) schools had a prevalence ranging between 10% and 24%, whilst the remaining 29 schools (11.0%) had a prevalence below 10%. As shown in Figure 4, most of the villages meeting the sustaining control prevalence range (i.e. 10-24%) that were ultimately selected (n = 75) are located in the Guemon region. The schools were randomly assigned to three intervention arms using a computer-based randomisation procedure conducted by an independent statistician.

### Data collection in the main study

#### Collection of stool samples and administration of a questionnaire

At the beginning of the main activities of the sustaining *S. mansoni* control study in the western part of Côte d’Ivoire, a small designated team informed district and village authorities and children’s parents. Detailed information was provided about the forthcoming cross-sectional parasitological and questionnaire surveys. Each year, shortly before the annual parasitological survey, teachers will be re-informed about the purpose and procedures of the study. Teachers assist by preparing class lists, including name, age, sex and school grade. In each school, children aged 9–12 years are enrolled from grades 2–6 until 100 pupils will be reached. Moreover, at baseline and in the final year of the study, 100 pupils will be randomly selected from the class list of grade 1 and their age will be recorded.

Before sample collection commences, the study is explained in lay terms to the selected children and they are invited to provide three stool samples over consecutive days. Stool samples are collected in 125 ml plastic containers. Children are asked to return the containers within an hour, filled with an apricot-sized portion of their own stool. We collect the containers and label them with individual IDs. The stool collection procedure is repeated over three consecutive days. Children without written consent from their parents and those who are unable to produce at least two stool samples are withdrawn, but they receive praziquantel treatment as the other children in the same village. For final analysis those children who have written consent and results from at least four Kato-Katz thick smears are included.

Upon visiting the school by our team, the community leader is contacted and interviewed with a pre-tested questionnaire to record characteristics of demography, main activities, health system, water contact sites, and sources of water and sanitary facilities in the village. Demographic information includes the number of households and total population. The health system is characterised by accessibility to health infrastructures, and availability of praziquantel against schistosomiasis and artemisinin-based combination therapy (ACT) against malaria. Information pertaining to water contact sites comprises the number of stagnant and non-stagnant freshwater bodies. The questionnaire also allows determining which type of water the population uses for drinking, bathing and cleaning. We also assess which type of sanitary facilities the population uses (i.e. pit latrine, ventilated improved pit latrine, toilet or any other kind of facilities).

Overall, the plan is to collect 22,500 stool samples from children aged 9–12 years and 7,500 stool samples from first-grade children at baseline (year 1) and at the end of the study (year 5). In years 2, 3 and 4, a total of 15,000 stool samples will be collected from children aged 9–12 years. Children on “drug holidays” will not be subjected to stool examination.

#### *Laboratory procedures to assess*S. mansoni *infection*

Fresh stool samples are transferred to central laboratories at the hospitals of Bangolo, Biankouma, Danané, Douékoué, Guiglo, Kouibly and Man. The stool samples are subjected to the Kato-Katz method [32, 33]. In brief, duplicate Kato-Katz thick smears are prepared from each sample using standard 41.7 mg templates. After a clearing time of at least 60 min, the thick smears will be examined under a microscope by experienced laboratory technicians. Eggs of *S. mansoni* and soil-transmitted helminths (*A. lumbricoides*, hookworm and *T. trichiura*) are counted and recorded for each species separately. For quality control, 10% of the slides are randomly selected and re-examined by a senior microscopist. The results are compared with the results of the first examination by the team. Slides identified with discrepant results (e.g. *S. mansoni* egg-positive *vs.* egg-negative or difference of *S. mansoni* egg counts of more than 20%) are re-examined until agreement has been reached. Given the large number of slides processed in the eligibility survey, parts of the slides were transferred to Abidjan and were examined microscopically within a maximum of 3 months after stool collection. All record sheets will be transferred to the Université Félix Houphouët-Boigny, where data entry, cleaning and database management take place.

#### SBT and assessment of coverage

District and community medical personnel are associated with the SBT in Côte d’Ivoire. Teachers and community health workers are trained to sensitize the communities, to administer drugs to the children and to monitor adverse events. Different sensitization tools are implemented such as radio and television announcements, along with other IEC strategies.

During SBT, praziquantel is administered by trained teachers to all children attending school. Children in all schools in the community are treated, even if the school is not among the 75 schools where children are being tested. Efforts are made to reach out to non-enrolled children to enhance treatment coverage.

Treatment is supervised by physicians and implemented by trained school teachers. Praziquantel tablets are delivered using a WHO dose pole [40]. Children are monitored for adverse events for 4 hours after treatment and, if need be, appropriate medical action is taken. Treatment will be led by the PNL-SGF, and supported by staff from the Programme National de Santé Scolaire et Universitaire (PNSSU). Financial support to facilitate treatment is provided by SCORE, while praziquantel tablets are supplied by the Schistosomiasis Control Initiative (SCI).

#### Data collection, management and statistical analysis

In the eligibility and baseline surveys, data are collected on paper form, but then, starting at year 2, smartphones have been utilised for data collection in the field and laboratory. Data cleaning and management is done by a designated database manager (PKY) at the Université Félix Houphouët-Boigny in Abidjan. Demographic and parasitological data directly entered in smartphones are uploaded to a database maintained on a central server (Open Data Kit) hosted by the SCORE secretariat at the Task Force for Global Health in Atlanta, United States of America. Data from questionnaire records will be entered in Microsoft Excel (2010 Microsoft Corporation). Statistical analyses will be carried out in STATA version 12 (StataCorp.; College Station, TX, USA). The primary outcome will be the change in prevalence and intensity of *S. mansoni* infection in the cohort of 9- to 12-year-old children over the four years of intervention. For each year, prevalence and infection intensity data will be calculated as described below. The results from the different study arms will be compared on an annual basis and at the end of the 4-year intervention period.

Each child with at least one *S. mansoni* egg identified in at least one of the Kato-Katz thick smears will be considered as positive. Eggs per gram of stool (EPG) will be determined for each child by calculating the arithmetic mean *S. mansoni* egg counts from all Kato-Katz thick smear readings and by multiplication with a factor 24. The *S. mansoni* infection intensity will be categorised according to WHO guidelines into light infection (1–99 EPG), moderate infection (100–399 EPG) and heavy infection (≥400 EPG) [22].

School attendance rate will be documented throughout the study. The treatment coverage will be determined by (i) calculating the percentage of pupils treated among the total children registered in the school and (ii) calculating the percentage of school-aged children not attending school who are treated according to data from community health workers.

To guarantee the privacy of individuals, a separate and confidential file will be kept, detailing names against ID numbers. All data will be stored in purpose-built MS Excel files with no names but only ID numbers and will be kept by the data manager at the Université Félix Houphouët-Boigny. A safety copy will be stored in a secured locker. Only authorized persons will have access to the data within the context of the project, and the data will be backed-up regularly and safely. In addition, all work stations of data entry clerks will be protected by case-sensitive passwords and there will be no sharing of any account or password information between staff and other individuals not concerned with the project. When discussing or showing the results of analyses in public venues, the information will always be reported at an aggregate level so that individual participants cannot be identified.

### Protocol review and ethical clearance

The study protocol has been approved by the institutional research commissions of Swiss TPH in Basel and CSRS in Abidjan. Ethical approval was obtained from the ethics committees in Basel (reference no. EKBB 279/10; Basel, 21 October 2010) and Côte d’Ivoire (reference no. 1994 MSHP/CNER; Abidjan, 5 May 2010). The trial is registered at controlled-trials.com (identifier: ISRCTN99401114; date assigned: 12 November 2014). Informed consent is obtained from parents or legal guardians of all pupils involved in the study. Children are treated with praziquantel (40 mg/kg) using a dose pole [40] in the frame of SBT. Efforts will be made to reach out to non-enrolled children.

The results of this study may be published, but subjects’ names or identities will not be revealed. Records will remain confidential and the results of tests will be codified to prevent association with participants’ names. Data entered into computerized files will be accessible only by authorized personnel directly involved in the study. Subject-specific information will be provided to medical personnel only with the subject’s permission.

## Discussion

Human schistosomiasis is a chronic and debilitating disease responsible for an estimated global burden of 3.3 million disability-adjusted life years [41]. In Africa, 230 million people currently require preventive chemotherapy [4]. The goal set by the WHO for the year 2020 is to treat at least 75% of school-aged children at risk of schistosomiasis with praziquantel as the only drug [7]. Various partners, institutions and pharmaceutical companies have agreed in the London Declaration of 2012 that they will contribute to achieving this goal with donation of praziquantel and other support to facilitate and sustain drug administration at large scale [42]. The SCORE study described here will provide an evidence-base for programme decisions about the type and frequency of preventive chemotherapy that is required to sustain control of schistosomiasis mansoni in areas where the baseline prevalence of infection ranges between 10% and 24%. The study will show for well characterised settings across Africa which treatment scheme (yearly treatment of school-aged children, or treatment interspaced by holidays) will yield the best result and at what cost. Moreover, factors that determine the effectiveness of large-scale deworming will be identified. These factors will help to develop measures of force of transmission that can be utilised to make decisions about the most cost-effective means of lowering the prevalence and intensity of *Schistosoma* infection and the force of the transmission in a given setting. The data generated might shape future treatment schedules to sustain the control of schistosomiasis at low level elsewhere in sub-Saharan Africa and perhaps in Asia and Latin America where schistosomiasis also remains endemic, in preparation for a move to eliminate this disease in suitable locations.

In western Côte d’Ivoire, infections with *S. mansoni* are common [17, 24, 34] but until the mid-1990s, the extent of endemicity was not well understood. Some control efforts had been implemented in the 2000s, but due to a decade-long socio-political crisis, control had been interrupted [28, 39]. The large eligibility study conducted to select 75 villages with a moderate *S. mansoni* prevalence (10-24%) clearly revealed that *S. mansoni* in the western part of Côte d’Ivoire is rampant; among 264 schools screened, 157 (59.5%) had a *S. mansoni* prevalence above 24%, while 78 schools (29.5%) were in the desired prevalence range of 10-24% and only 29 schools (11.0%) had a prevalence below 10%. The eligibility survey employed an insensitive diagnostic approach (i.e. duplicate Kato-Katz thick smears based on a single stool sample). Had more intensive sampling and a more sensitive diagnostic method been employed (e.g. three stool samples subjected to triplicate Kato-Katz per stool sample or a point-of-care circulating cathodic antigen (POC-CCA) urine cassette test), a much higher overall prevalence of *S. mansoni* would have been found [43–47].

In order to respect World Health Assembly (WHA) resolution 54.19 endorsed in May 2001, which emphasises preventive chemotherapy targeting school-aged children to control morbidity [48], and in view of a more ambitions WHA resolution 65.21 put forth in May 2012, the new declared goal is to move from morbidity control to elimination of schistosomiasis [33, 49]. Hence, authorities of Côte d’Ivoire have established the PNL-SGF in June 2007 [28]. Hand-in-hand with the SCORE sustaining schistosomiasis control operational research study reported here, the national schistosomiasis control programme was reinforced, with additional support from SCI. This study will provide data that may be used by SCORE, SCI, WHO and other partners to deploy the best tools and strategies to control morbidity due to schistosomiasis, and thus contribute to the reduction of poverty in schistosomiasis-endemic countries.
